# Thrombotic microangiopathy in oncology – a review

**DOI:** 10.1016/j.tranon.2021.101081

**Published:** 2021-04-13

**Authors:** Patrícia Valério, João Pedro Barreto, Hugo Ferreira, Teresa Chuva, Ana Paiva, José Maximino Costa

**Affiliations:** a Nephrology Department, Setúbal Hospital Center, Portugal Rua Camilo Castelo Branco 175, 2910-549 Setúbal, Portugal; b Laboratory Diagnosis Department, Portuguese Oncology Institute of Porto, Portugal Rua Dr. António Bernardino de Almeida, 4200-072 Porto, Portugal; c Nephrology Department, Portuguese Oncology Institute of Porto, Portugal Rua Dr. António Bernardino de Almeida, 4200-072 Porto, Portugal

**Keywords:** Cancer, Haematological malignancies, Hematopoietic stem cell transplantation, Onco-nephrology, Thrombotic microangiopathies

## Abstract

Thrombotic microangiopathy is a syndrome triggered by a wide spectrum of situations, some of which are specific to the Oncology setting. It is characterized by a Coombs-negative microangiopathic haemolytic anemia, thrombocytopenia and organ injury, with characteristic pathological features, resulting from platelet microvascular occlusion.

TMA is rare and its cancer-related subset even more so. TMA triggered by drugs is the most common within this group, including classic chemotherapy and the latest targeted therapies. The neoplastic disease itself and hematopoietic stem-cell transplantation could also be potential triggers.

Evidence-based medical guidance in the management of cancer-related TMA is scarce and the previous knowledge about primary TMA is valuable to understand the disease mechanisms and the potential treatments.

Given the wide spectrum of potential causes for TMA in cancer patients, the aim of this review is to gather the vast information available. For each entity, pathophysiology, clinical features, therapeutic approaches and prognosis will be covered.

## Graphical abstract

**Figure:**
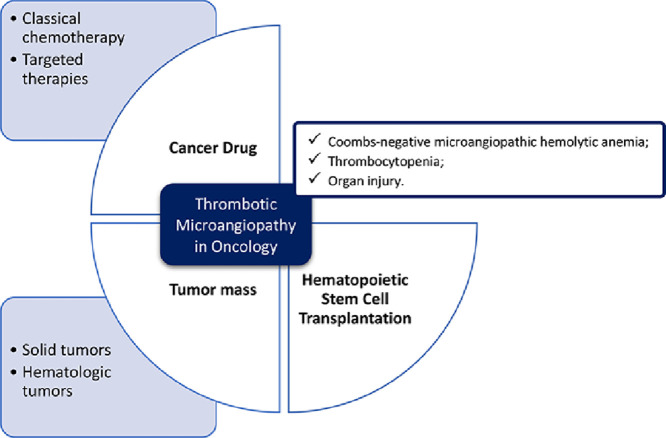

## Introduction

Thrombotic microangiopathy (TMA) is a syndrome triggered by a wide spectrum of situations, with common pathological and clinical features [1]. It was first described in 1924, by Moschcowitz, who reported a fatal case of a 16-year-old-girl with fever, weakness, transient focal neurologic symptoms, severe thrombocytopenia, and microangiopathic haemolytic anemia (MAHA) [2]. Later, in 1947, the term Thrombotic Thrombocytopenic Purpura (TTP) was used for the first time by Singer K. et al. [3]

Classically, the most recognized TMA are TTP and Haemolytic Uremic Syndrome (HUS), which can be acquired or hereditary. A deficit in the activity of ADAMTS13, a von Willebrand factor (VWF) cleaving protease, is the hallmark of TTP. In its absence, ultra-large VWF multimers accumulate, leading to platelet aggregation, endothelial damage and microvasculature thrombosis. HUS usually results from infection by bacterial species that produce Shiga toxin, in particular *Escherichia coli* O157:H7 (STEC). The remaining cases, caused by dysfunction of the complement alternative pathway, are designated atypical HUS (aHUS) [1].

The secondary forms of TMA include an extensive range of causes, varying from infections to cancer, pregnancy, malignant hypertension, drugs, solid or hematopoietic transplantation, and autoimmune or metabolic diseases, etc. Despite the varied pathophysiology the presence of endothelial damage leading to microvascular ischemia is the hallmark of TMA [1,[4], [5], [6], [7], [8]].

Clinically, the syndrome is characterized by [1,4,6]:

• A Coombs-negative MAHA characterized by elevated serum lactate dehydrogenase (LDH) level, undetectable or markedly decrease serum haptoglobin and the presence of schistocytes on a peripheral blood smear (although a non-obligatory criteria).
• Thrombocytopenia;
• Organ injury: kidney disease, neurologic symptoms and gastrointestinal manifestations, amongst others; kidney involvement may include acute kidney injury (AKI), proteinuria or hypertension (HTN);
• Normal coagulation.

The pathologic findings of TMA in kidney biopsy include eosinophilic hyaline thrombi, mainly composed by platelet aggregates - a signal of vascular injury in response to endothelial injury [6,9].

TMA investigation will only be pursued if a complete anamnesis and physical examination allows for a high grade of suspicion. This is an important aspect due to the fact that TMA is the presentation of many different diseases, with a wide differential diagnosis that demands a multidisciplinary approach [4].

TMA is a well-recognized complication in Oncology setting, either as a consequence of cancer or of its treatments. It is a rare but potentially life-threatening complication, in a group of patients whose prognosis is already worse than that of the general population.

Notwithstanding the rarity of TMA, early recognition and treatment are essential to minimize its burden, including dialysis-dependent chronic kidney disease (CKD). There are a few therapeutic options, with support therapy and drug withdrawal being the most widely accepted.

Plasmapheresis plays a central role in TTP and remains an invaluable asset in other forms of TMA, at least in particular situations. This technique replaces patient plasma with donor plasma, allowing the removal of potential endothelial damaging agents or autoantibodies, and the replacement of certain molecules essential for endothelial function, such as ADAMTS13 [10].

There are some case reports with the use of rituximab (an anti-CD20 monoclonal antibody), and a few studies about complement inhibition (such as eculizumab, a monoclonal antibody against complement factor C5) in specific situations [6, [11], [12], [13]].

However, none of these therapies can be formally recommended, since there are no randomized controlled trials available yet.

Given the wide spectrum of potential causes for TMA in cancer patients, the aim of this review is to gather the vast information available. For each entity, pathophysiological mechanisms, clinical features, therapeutic approaches and prognosis will be covered.

## Cancer drug-induced TMA

The first cancer drug recognized as a cause of TMA was Mitomycin C (MMC), but currently there is a fast-growing list of potential TMA triggers [1,5,6,14].

Drug-induced TMA (DiTMA) can be either dose- and time-dependent or non-dose-related (idiosyncratic reactions). The latter situation should be suspected when there is an abrupt onset of symptoms that recur with drug administration, and it is mostly caused by drug-dependent antibodies that react with platelets and endothelial cells. Dose/time-dependent situations are usually related to a slowly progressive kidney injury in consequence of TMA [1,6]. Mechanisms that lead to complement activation or ADAMTS13 deficiency have also been suggested [6,14,15]. Independently from the trigger, drug-induced endothelial injury is assumed as the initiating event in DiTMA, but the specific mechanism remains unclear for many drugs [1,6,15].

Several clues may point to DiTMA: the timing of TMA development; partial or complete recovery after drug withdrawal; and previous reports of association with drugs of the same class [1,6].

The wide range of drugs associated with cancer DiTMA implies different clinical presentations, including the degree on kidney function impact, reversibility and mortality [6,14,15]. As to the effects in kidney function, classically, cancer DiTMA can be classified in 2 categories (Table 1) [6]:

  • Type 1: its agents include classical chemotherapy drugs; it could lead to CKD, increased morbidity and mortality;
  • Type 2: it is usually presented as a kidney-limited TMA with long-term drug exposure, but also relatively stable kidney function and frequent recovery; its agents include the most recent cancer-drugs (targeted therapies).

**Table 1:** Type 1 and type 2 DiTMA cancer drugs; VEGF - vascular endothelial growth factor. Adapted from [6].

	Type 1 agents	Type 2 agents
**Characteristic agents**	2- Mitomycin C 2- Gemcitabine 2- Platinum salts 2- Proteasome inhibitors	2- Anti-VEGF 2- Tyrosine-kinase inhibitors 2- Proteasome inhibitors
**Onset**	Delayed: usually 6 to 12 months after initiation	At any time
**Clinical**	Permanent and irreversible; classical TMA syndrome	Possibly reversible after interruption; hematologic manifestations only in some patients
**Effect of rechallenge**	High probability of recurrence	Considered to be relatively safe
**Pathology**	Arteriolar and glomerular capillary thrombosis	Almost exclusive glomerular thrombosis
**Prognosis**	High incidence of mortality and CKD requiring haemodialysis	Patient and kidney survival rates are excellent after drug stop

The Common Terminology Criteria for Adverse Events (CTCAE), created by National Cancer Institute of United States of America (NCI), are the criteria that allow the standardization of adverse effects classification of drugs used in cancer therapy. The last version (5.0), defines TMA as an acute or subacute disorder characterized by MAHA, thrombocytopenic purpura, fever, kidney dysfunction and neurological abnormalities (e.g., seizures, hemiplegia, visual disturbances) [16]. It comprises 3 clinically relevant severity grades:

■ Grade 3: Laboratory findings with clinical consequences (e.g., kidney insufficiency, thrombocytopenic purpura);
■ Grade 4: Life-threatening consequences (e.g., central nervous system hemorrhage/thrombosis/embolism or kidney failure);
■ Grade 5: Death.

Besides the aforementioned hyaline *thrombi*, some patients with DiTMA show nonthrombotic features on kidney biopsies, namely endothelial swelling and denudation, mesangiolysis, double contours of the glomerular basement membrane, intramural fibrin, myxoid intimal thickening or concentric myointimal proliferation (onion-skinning), suggesting a sub-acute or chronic process [9].

### Classical chemotherapy

#### Mitomycin C

MMC is an antibiotic that works as a cell-cycle specific alkylating agent, still used in bladder cancer [17]. MMC-induced TMA was first described in 1971, becoming the prototype of TMA caused by classical chemotherapy [18].

It could occur immediately after administration or until 15 months after the last dose. Most cases occur after 6 months, with a cumulative dose that exceeds 50 mg/m^2^ [18,19].

The pathogenesis of MMC-induced TMA involves direct endothelial toxicity [18,20], although immune-mediated reactions have also been described [21,22].

The clinical presentation is usually characterized by a slowly progressive kidney dysfunction, HTN and a bland urine sediment [23,24]. Some patients develop a noncardiogenic pulmonary edema associated with an acute respiratory distress syndrome (ARDS), probably due to pulmonary endothelial damage [21,23,25,26].

Nephrotoxicity may be avoided by limiting the total cumulative dose. Drug withdrawal and supportive care are the only therapeutic approaches available. The response to plasmapheresis is poor, and red blood cells (RBC) transfusions could exacerbate TMA [21,23]. Some reports suggest improvement in survival and kidney function with rituximab [24,27] and eculizumab [28,29].

The prognosis is poor, with a mortality rate of almost 75% at 4 months, mainly in the cases with pulmonary involvement [23,25,26]. Survivors often suffer from some degree of kidney dysfunction [22,23].

#### Gemcitabine

Gemcitabine is a pyrimidine analogue, acting as a cytotoxic agent, that rarely triggers TMA (<1%). However, most recent case series present higher incidences, as indications for the drug grow [13,30].

Gemcitabine-induced TMA can occur immediately after administration, but the median time is approximately 8 months [31]. An average cumulative dose above 20 g/m^2^ has been associated with TMA [13,30], but concomitant use of other drugs could lower this threshold [31,32].

Gemcitabine is the chemotherapeutic drug with the strongest evidence of toxicity by both direct endothelial damage and deposition of immune complexes [32,33].

Gemcitabine-induced TMA clinically presents as a predominantly kidney-limited disease, with AKI in almost every patient, frequently requiring haemodialysis. Proteinuria and haematuria may also be present. New-onset or exacerbation of HTN could occur several months before the diagnosis [30,31].

Treatment is based on immediate and permanent drug cessation [30,34] and recurrence has been described with repeated exposure [32]. RBC and platelets transfusions exacerbate TMA and should be avoided. Despite the pathological mechanism, response to plasmapheresis is poor [30,34,35]. Few case reports document successful use of rituximab [11,36,37] and eculizumab [35,38,39].

The prognosis is poor, with a high mortality rate (40–90%) and kidney dysfunction is liable to persist in recovered patients [30].

#### Platinum-based drugs

Platinum-based drugs are alkylating agents frequently used in Oncology. Nephrotoxicity, whereas uncommon, is a major adverse effect [40].

Cisplatin-induced TMA is rare, but it has been described in several case reports, alone or in association with other chemotherapeutic agents [41], [42], [43]. The exact mechanisms are not completely understood, although there is evidence of direct toxicity to endothelial cells, with subsequent complement activation [41,44]. Patients present systemic features of TMA, in association with AKI [42,45,46]. At least one case reported successful use of eculizumab, but the patient also presented a complement protein mutation [41]. Plasmapheresis showed inconsistent results [47,48].

Oxaliplatin and carboplatin are more recent platinum analogues, rarely associated with TMA [6]. Some reports of oxaliplatin describe an antibody-mediated TMA that occurs suddenly, within minutes after subsequent drug exposure [49]. An Italian report used eculizumab with success in a patient with a complement protein mutation [50].

Carboplatin has very few cases reported, mostly in coadministration with drugs like gemcitabine, docetaxel or trastuzubam [51], [52], [53].

#### Pegylated liposomal doxorubicin

Doxorubicin is an anthracycline that acts as a topoisomerase II inhibitor. Pegylated liposomal doxorubicin (PLD) is commonly used in recurrent ovarian cancer. This formulation markedly prolongs the half-life in the vascular compartment and limits its adverse effects [54,55].

Kwa et al. [56] reported 3 patients with a biopsy-proven kidney TMA following years of PLD administration and high cumulative doses (880 to 1445 mg/m^2^). The syndrome was characterized by AKI, non-nephrotic proteinuria and HTN, without anemia or thrombocytopenia. However, one of them was simultaneously treated with bevacizumab and, several years before, the 3 patients were treated with platins [54,56].

The treatment of PDL-induced TMA relies on supportive care and drug withdrawal, most times resulting in TMA regression [56].

#### Bleomycin

Bleomycin, an antibiotic that inhibits DNA synthesis, is frequently used in combination with platins in several cancers [42,46]. Endothelial damage is a known effect of bleomycin, with case reports describing Raynaud's phenomenon, digital infarcts and pulmonary toxicity. However, in all reports of bleomycin and TMA, there is a concurrent drug that could trigger TMA by itself [42,43,46]. There is no evidence to define whether TMA is caused by a synergic mechanism. At least one case report reported successfully on the use of eculizumab [57].

#### Docetaxel

Docetaxel is a taxane derivative that stabilizes microtubules, used in several solid cancers. There are a few cases assuming docetaxel-related TMA. Nevertheless, the presence of an alternative potential TMA trigger, namely another drug [51,58] or a metastatic cancer [59], was frequent.

#### Pentostatin

Pentostatin is a purine analogue, acting as a cytotoxic agent, used in hairy cell leukaemia and some T-cell lymphomas. TMA is rare and the suggested mechanism is direct toxic endothelial damage [24]. Mayo Clinic [60] reported 4 cases associated with chronic lymphocytic leukaemia, characterized by AKI, MAHA and thrombocytopenia.

### Targeted therapies

NCI defines targeted therapies as “*drugs or substances that block growth and spread of cancers by interfering with specific molecules involved in tumor growth and progression*”. In the last decade, there has been a revolution in the development of these anticancer drugs. TMA is a potential adverse effect when the targeted molecule interferes with endothelial homeostasis [61].

#### VEGF inhibitors

Vascular Endothelial Growth Factor (VEGF) is critical in blood vessel growth. Its inhibition limits endothelial proliferation, with reduction in tumor blood supply, a mechanism used to treat several malignancies [62,63]. VEGF inhibition can be achieved in 2 ways: antibody-mediated binding of the ligand (bevacizumab and aflibercept) or receptor inhibition (tyrosine kinase inhibitors – TKIs) [5,6].

In a healthy kidney, VEGF is produced by podocytes and regulates the integrity and function of the actin skeleton of endothelial cells. This makes glomerular endothelium particularly susceptible to VEGF inhibition, leading to loss of the fenestrated endothelium, microvascular injury and, eventually, TMA [63,64].

The clinical spectrum of kidney adverse effects of VEGF inhibitors (VEGFi) is composed by a new-onset or exacerbation of HTN, proteinuria (sometimes in nephrotic range) and TMA with severe AKI. Proteinuria is described as an indirect sign of the anticancer effect of this drug. Kidney-limited TMA (type 2) is the main phenotype reported, with only mild anemia or thrombocytopenia [6,63,64].

Several authors describe VEGFi-induced TMA as a “*preeclampsia-like syndrome*” [62], [63], [64], [65], with several clinical and histopathological features common to preeclampsia, but that could occur in non-pregnant individuals. preeclampsia is characterized by new onset HTN with significant end-organ dysfunction, occurring after 20 weeks of gestation, in which the kidney is frequently involved, with proteinuria or oliguric kidney injury. preeclampsia pathophysiology is not completely understood, but relies on endothelial damage due to VEGF dysregulation and recent evidence shows an imbalance between pro-angiogenic and anti-angiogenic molecules: excess of fms-like tyrosine kinase 1 (sFlt-1), a soluble receptor of VEGF, leads to a reduced VEGF effect on glomeruli and damage to the podocytes, with reduced expression of nephrin and synaptopodin (proteins essential for the actin skeletal functions) [63,64,66]. Higher levels of sFlt-1 were already detected in patients with TMA induced by VEGFi [64,65]. Additionally, there are common clinical characteristics to both preeclampsia and VEGFi-induced TMA, namely transaminitis (it is unknown if this is due to drug indirect or direct effect), resolution after delivery or drug withdrawal, and endotheliosis and podocytes foot processes effacement on kidney biopsy [63], [64], [65].

A French case series reported mild to severe acute tubular necrosis [65] in some patients, in addition to TMA, suggesting a common pathological pathway triggered by VEGF inhibition. In this series, kidney adverse effects were dose-related and clinical presentation resembled a rapidly progressive kidney failure. Isolated TMA allows to continue the drug under tight surveillance, in the absence of an alternative cancer therapy. However, the presence of ATN is a formal indication to stop the drug immediately [64].

Blood pressure control is essential and renin-angiotensin system blockers are preferred, since VEGF inhibition induces renin secretion through tissue ischemia [64,67]. Some case reports described the successful use of eculizumab, even while still administrating VEGFi [57,68]. In general, kidney prognosis is good with drug withdrawal [6].

#### Tyrosine kinase inhibitors

TKIs are drugs that inhibit the intracellular signaling pathways of numerous tyrosine kinases receptors, including those for: VEGF (VEGFr), platelet-derived growth factors (PDGFr), epidermal growth factor (EGFR), etc. [6,15].

Several TKIs are related to DiTMA, but the majority of case reports refer to sunitinib, a multitargeted TKI that inhibits VEGFr. In fact, this is the most well-known mechanism for TKIs-induced TMA [62,67,[69], [70], [71]].

Other multitargeted TKIs, including some with no effect on VEGFr, have also been associated with DiTMA (e.g., imatinib, sorafenib). Immune mediated and direct endothelial toxicity mechanisms are probably involved [62,72,73].

TKIs are less associated with TMA when compared to VEGFi. However, combination therapy with these 2 drug classes resulted in a more severe form of TMA [6,71,74].

Withdrawal of the offending drug and blood pressure control are the cornerstone of treatment, showing good results [6].

#### Proteasome inhibitors

The ubiquitin proteasome pathway is critical in the cell cycle, destroying targeted proteins. Bortezomib, carfilzomib and ixazomib act through proteasome inhibition, preventing the degradation of pro-apoptotic factors. Presently, they are used in several monoclonal gammopathies [56], including multiple myeloma [13,[75], [76], [77]].

Kidney disease could be a complication of monoclonal gammopathy, including cast nephropathy, glomerular diseases and electrolyte disorders. It is essential to distinguish proteasome inhibitors (PIs) kidney toxicity from complications of gammopathies [78].

VEGF inhibition is a potential pathological pathway in PIs-induced TMA, because they affect VEGF production through nuclear factor kappa B (NF-κB) inhibition [75], [76], [77]. Immune-mediated and dose-dependent toxicity have also been proposed [75, 79]. Moore et al. [79] reported a patient with a syndrome similar to TTP with autoantibodies directed to ADAMTS13.

Most reports implicated bortezomib and carfilzomib, describing a systemic syndrome with MAHA, thrombocytopenia and AKI. Kidney injury was partially reversible with drug discontinuation and supportive care. TMA recurrence was observed with drug rechallenge [15,80]. More recent reports support a causal association of ixazomib with DiTMA, suggesting this is a class adverse effect [80], [81], [82].

Some authors reported favourable results using eculizumab in carfilzomib-induced TMA. In those cases, ADAMTS13 activity was normal, suggesting a pathophysiology similar to aHUS, with complement overactivation [83]. One additional report refers to a patient with a complement mutation [84]. As expected, plasmapheresis showed no clear benefit in this setting [75].

#### Immune checkpoint inhibitors

Immunotherapy is an emerging strategy to treat solid and hematologic malignancies, improving overall survival. Immune checkpoint inhibitors (CPIs) are part of this class of drugs, consisting of monoclonal antibodies that target inhibitory receptors expressed on T cells [85].

Recently, there have been some reports of CPIs-induced TMA with ipilimumab and nivolimumab. Despite the temporal correlation, true causality is difficult to establish, since the reported cases presented confounding factors, namely metastatic malignancy and other drugs related with TMA [85].

The mechanisms remain unclear, and additional cases are needed before TMA can be reliably attributed to CPIs.

#### Other monoclonal antibodies

Monoclonal antibodies are used in a wide variety of diseases, with an increasing number of drugs targeted to Oncology. Cetuximab and trastuzumab have been implicated in DiTMA in case reports. Additional cases are essential to attribute TMA to these drugs [86, 87].

Table 2 summarises cancer drugs-induced TMA.\

**Table 2:** Summary of cancer DiTMA. For drugs with weaker evidence, the reference is cited. Abbreviations: AKI – acute kidney injury; ARDS – acute respiratory distress syndrome; BCR-ABL – Philadelphia chromosome; CPIs – checkpoint inhibitors; EGFR – epidermal growth factor receptor; HTN – hypertension; MMC- mitomycin C; PDGFr – platelet-derived growth factors receptor; PIs – proteasome inhibitors; PLD – pegylated lysosomal doxorubicin; TTP – thrombotic thrombocytopenic purpura; TKIs – tyrosine kinase receptors; VEGF – vascular endothelial growth factors; VEGFr – VEGF receptor. Adapted from [5].

Drugs		Mechanism	Clinical features	Treatment
MMC		■ Direct endothelial damage; ■ Immune mediated damage: ■ Prostacyclin inhibition.	■ HTN, AKI, ARDS (rare); ■ Six to 15 months after last dose; ■ Dose-dependent: cumulative dose >40–50 mg/m^2^; ■ Kidney dysfunction may be permanent.	■ Drug cessation; ■ Rituximab; ■ Eculizumab.
Gemcitabine		■ Direct endothelial damage; ■ Drug-dependent antibodies.	■ Predominantly kidney-limited ■ Severe AKI, requiring haemodialysis - dysfunction may persist; ■ Mostly delayed after last dose; ■ Recurrent episodes with repeated exposure; ■ Dose-dependent: median cumulative dose >20 g/m^2^.	■ Drug cessation; ■ Rituximab; ■ Eculizumab.
Platinum-based drugs	Cisplatin	■ Direct endothelial damage.	■ Systemic TMA; ■ AKI.	■ Drug cessation; ■ Eculizumab?
	Oxaliplatin [49,50]	■ Drug-dependent antibodies?	■ AKI → *TTP like syndrome*; ■ Suddenly after sub-sequent drug administration.	
	Carboplatin [53,51,52]	*Unknown – coadministration with other potential triggers.*	*Very rare*	■ Drug cessation.
PLD [54,56]		*Unknown – coadministration with other potential triggers.*	■ Kidney-limited TMA: AKI, non-nephrotic proteinuria, HTN; ■ Dose-dependent: cumulative doses >880–1445 mg/m^2^.	■ Drug cessation.
Bleomycin [42,43,46,57]		■ Direct endothelial damage? *Other potential triggers.*	■ Systemic TMA; ■ AKI; ■ Non-cardiac pulmonary edema.	■ Drug cessation; ■ Eculizumab?
Docetaxel [51,58,59]		*Unknown;* *Other potential triggers.*	■ Systemic TMA; ■ AKI.	■ Drug cessation.
Pentostatin [24,60]		■ Direct endothelial damage? *Other potential triggers.*	■ Systemic TMA; ■ AKI, sub-nephrotic proteinuria and HTN.	■ Drug cessation.
VEGF inhibitors – antibody-mediated binding of the ligand: ■ Bevacizumab; ■ Aflibercept.		■ Indirect endothelial damage – VEGF pathway inhibition	■ Dose-dependent; ■ Kidney-limited TMA: AKI, proteinuria or HTN. “preeclampsia*-like syndrome*”	■ Drug cessation, with good outcomes; ■ Eculizumab.
TKIs – multitargeted receptor inhibition: ■ Sunitinib: VEGFr, PDGFr; ■ Imatinib: BCR-ABL receptor; PDGFr; ■ Sorafenib: VEGFr, PDGFr;		■ VEGF pathway inhibition; ■ Other mechanisms? - unknown.	“preeclampsia*-like syndrome*”	
PIs: ■ Bortezomib; ■ Carfilzomib; ■ Ixazomib [82,81].		■ Direct endothelial damage; ■ Indirect endothelial damage: ­ VEGF pathway inhibition; ­ Autoantibodies directed to ADAMTS13.	■ Dose-dependent?; ■ Systemic TMA; ■ AKI and HTN.	■ Drug cessation, with good outcomes; ■ Eculizumab?
CPIs: ■ Ipilimumab; ■ Nivolimumab.		*Not fully understood.*	■ HTN; ■ AKI.	■ Drug cessation; ■ Rituximab?
Monoclonal antibodies: ■ Cetuximab: anti-EGFR; ■ Ramucirumab: anti-VEGFr2.		■ VEGF pathway inhibition; ■ Other mechanisms? - unknown.	■ Nephrotic syndrome; ■ *“preeclampsia-like syndrome”* with anti-VEGFr.	■ Drug cessation, with good outcomes.

## Hematopoietic stem cell transplantation-induced TMA

Several hematologic and selected solid tumours are treated with HSCT. Since the 1980s, TMA has been a well-documented HSCT complication [8,[88], [89], [90]].

HSCT-induced TMA (HSCT-TMA) has a wide range of reported incidence (up to 80%), that may be explained by the retrospective nature of the studies, the simultaneous analysis of pediatric and adult populations and a lack of uniform diagnostic criteria. These are highly complex patients because of the procedure itself and its adverse effects, such as bone marrow ablation, toxic drug effects and immunological reactions against an allogenic graft. Consequently, a high level of suspicion is essential to make an early diagnosis [8,[88], [89], [90], [91], [92]].

### etiology and pathophysiology

HSCT-TMA pathophysiology is complex and not fully understood, but endothelial dysfunction is probably independent of ADAMTS-13 activity [8,89,93].

The clinical association with several risk factors is well established, probably with a synergic effect [93]. Those factors are: graft-versus-host disease (GVHD) [94], [95], [96], [97], [98]; calcineurin inhibitors (CNI) [90,94]; mammalian target of rapamycin inhibitors (mTORI) [95]; disseminated infections [91,98,99]; myeloablative and conditioning regimens, especially those with a high dose of busulfan and total-body irradiation (TBI) [88,95,96]; and HLA mismatch [90,96].

**Fig. 1:**
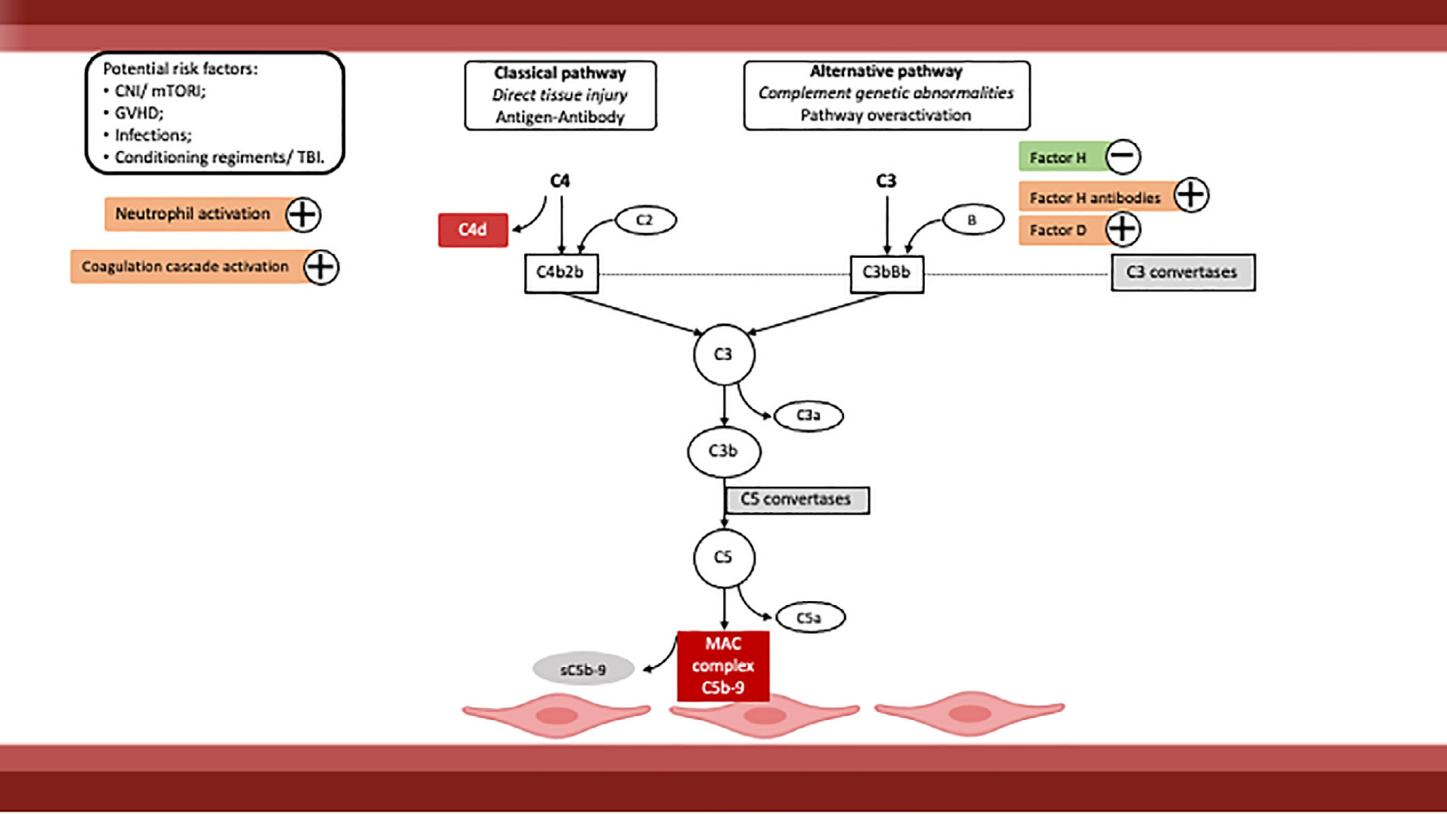
HSCT-TMA pathogenesis. Abbreviations: CNI, calcineurin inhibitors; GVHD, graft versus host disease; MAC, membrane attack complex; mTORI, mammalian target of rapamycin inhibitors; TBI, total body irradiation.

Recently, evidence of a central role of complement has emerged, with involvement of both classic and alternative pathways (Fig. 1) [100], [101], [102], [103], [104], [105], [106], [107], [108], [109]. In fact, some of the described risk factors also have the ability to activate complement pathways, including high doses of CNI/mTORI, viral infections and GVHD [102,110].

#### Role of graft versus host disease

GVHD is an epithelial cell disease mostly affecting the gastrointestinal tract, skin and liver, that has been identified as a major risk factor for HSCT-TMA [8,[94], [95], [96], [97]]. However, HSCT-TMA also occurs in patients with no GVHD [99].

Endothelial injury is also a feature of GVHD, with endothelial cells as the putative direct target of donor cytotoxic T lymphocytes [88,106,111]. Patients with acute GVHD after HSCT present high levels of plasma markers of endothelial injury, indicating a probable link between endothelial lesion and the development of both GVHD and HSCT-TMA [8,106,112].

An autopsy-based study showed that patients with acute GVHD had a higher probability of developing TMA, independently of CNI and mTORI use [97]. On the other hand, a series of 4 cases of biopsy-proven HSCT-TMA described a simultaneously acute and/or chronic GVHD in other organs [113].

These data suggest a link between HSCT-TMA and GVHD, possibly sharing a not completely understood pathological mechanism. Moreover, several authors propose an aggressive treatment of GVHD in patients with HSCT-TMA [88, 94].

#### Role of calcineurin inhibitors

CNI are commonly used in the prophylaxis of acute GVHD in allogenic HSCT. For years, HSCT-TMA has been causally linked to CNI and mTORI, despite the weak evidence [8].

Labrador et al. [94] reported no differences in the incidence of HSCT-TMA between patients receiving prophylaxis with cyclosporin+methotrexate versus tacrolimus+methotrexate, with the exception of those patients achieving higher serum tacrolimus levels. Another study by Shayani et al. [95] described an association between TMA and higher serum sirolimus levels, in a range believed to be nontoxic (>9,9 ng/mL). An autopsy cohort did not find correlation between CNI use and TMA [97].

In allogenic HSCT there is at least one study showing that TMA can occur in patients in which CNI and mTORI were not used [114].

TMA was also described in autologous HSCT, where CNI and mTORI are not used. In those cases, aggressive conditioning regimens may act as triggers for TMA, especially when platin-based drugs are required [115], [116], [117].

Currently, there is not enough information to guide clinical decisions in this context.

#### Role of complement

Complement system is part of innate immunity and its major function is to help antibody-triggered responses. There are three major pathways for complement activation with distinct triggers: the lectin pathway is triggered by repetitive patterns of carbohydrates on the surface of microorganisms; the classical pathway is initiated by antibodies; and the alternative pathway is constitutionally activated, being regulated by plasma (fluid-phase) regulators. Each one leads to C3 activation, with C3b target deposition, which starts the assembly of the membrane attack complex (MAC), the ultimate goal of complement – terminal pathway. The MAC leads to the lysis of microorganisms. Simultaneously, the products formed by each pathway will promote inflammatory response (e.g. C3a, C5a) [118].

Complement dysfunction, either hereditary or acquired, is recognized as part of the pathophysiology of several diseases, namely systemic lupus and aHUS. Measurement of complement molecules and functional assays (for example, CH50 for the classical pathway, AP50 for the alternative pathway) allow recognition of the overactivated pathway [93,118].

In the context of HSCT, there is growing evidence of complement involvement in TMA [92,93]. At least two prospective studies with HSCT-TMA patients showed an overactivation of terminal complement pathway, with higher levels of sC5b-9 associated to a worse prognosis [105,119,120].

C4d staining is used in the assessment of antibody-mediated rejection in kidney transplant biopsies and is a marker of the classical complement pathway activation. In a study with 20 children (8 of which had TMA), arteriolar C4d staining was more commonly found in patients with HSCT-TMA (75% versus 8%), which could represent complement activation by recipient- and donor-specific antibodies [8,107]. Additionally, significantly higher CH50 levels were described in HSCT-TMA patients [104,120].

Alternative complement pathway involvement has also been recognized. A recent study [121] showed higher levels of factor Ba, which presented an inverse relationship with estimated glomerular filtration rate (eGFR).

Evidence of genetically conditioned dysfunction in both the classical and alternative pathways has also emerged, associated with mutations in specific complement proteins and anti-factor H autoantibodies [107], [108], [109].

Likewise, multiple gene variants were associated with a genetic susceptibility to HSCT-TMA. These probably would not be significant in normal circumstances but may lead to complement activation under the extremely stressful situation of HSCT. This concept supports the “multi-hit hypothesis”, already described in TMA due to other causes, with multiple risk factors synergically inducing TMA within a favourable environment. This would explain why different individuals, even under the same circumstances, may show a wide range of different clinical evolutions [92,93,109]. Genetic susceptibility can also provide a good explanation for the poor response and survival to conventional therapy with plasmapheresis, in certain situations [109].

To add complexity to this issue beyond complement activation, Gavriilaki et *al*. recently suggested that neutrophil and coagulation cascade activation could also be of importance in HSCT-TMA pathophysiology [106].

However, the central role of complement is undeniable, and the available evidence identifies it as a targetable pathway, providing a foundation for the use of eculizumab. In the last years, several case reports and retrospective studies showed eculizumab efficiency in this context (see ahead) [100], [101], [102], [103], [104]. Lack of adequate response to complement blockage in some patients, on the other hand, suggests potential additional endothelial injury pathways, requiring more investigation [103].

To sum up, even if no etiological role has been recognized with certainty in patients with HSCT-TMA, it is clear the association between endothelial damage and complement overactivation, both in early and terminal pathways.

### Clinical presentation and patient evaluation

Immediately after HSCT there is a high risk of AKI, with risk factors attributable to the procedure and the drugs administrated. Serum creatinine levels will probably not provide a reliable estimation of GFR, because these patients may display large fluctuations in nutritional status, weight and muscle mass [91,97].

The most common clinical presentation is a subacute or chronic TMA that becomes apparent weeks after HSCT. Kidney manifestations include AKI, proteinuria, and HTN [8]. TMA should be suspected in patients who develop new-onset or exacerbation of HTN, requiring more than 2 anti-hypertensive drugs. Systolic HTN has been appointed as an early marker for TMA, particularly in children [8,95,105,122].

Although the kidneys are frequently affected, involvement of other organs has been reported, mostly the gastrointestinal tract and the lungs [91,115].

Besides the usual TMA workup, these patients require an active search for infections whenever sepsis is a possibility [91]. Mutations in complement proteins and anti-H factor autoantibodies should be investigated in selected patients [107].

A kidney biopsy would give precious diagnostic and prognostic information. However, it is not always feasible, mainly because of high bleeding risk [122]. The biopsy findings are similar to those of TMA by other causes. In rare cases, tubular reticular inclusions can be found, suggesting a high level of interferon activity, even in the absence of viral infection. Immunofluorescence is generally negative for immune complexes, although there may be unspecific fibrin deposition [8]. Autopsy studies have showed a poor correlation between clinical and histological findings [91,97].

### Diagnosis

Diagnosing HSCT-TMA is difficult and requires integration of clinical and pathologic data. These patients frequently develop MAHA, thrombocytopenia and kidney dysfunction in a post-transplantation period, due to innumerable reasons [89,91].

As pointed before, kidney biopsy is not always performed [97,122]. The difficulty of histologic diagnosis led to the development of non-invasive clinical criteria for HSCT-TMA diagnosis. The first ones were presented by the Bone Marrow Transplant Clinical Trials Network (BMT-CTN) [123] in 2005, followed by International Working Group (IWG) [124] in 2007. Cho et al. [125] conducted a retrospective analysis of those criteria with 672 allogenic HSCT recipients and highlighted their limitations; the concept of “probable TMA” was introduced, with analytical evidence of MAHA and thrombocytopenia, even in the absence of kidney or neurologic dysfunction. More recently, Jodele et al. [126] presented wider criteria that included kidney and/or neurological dysfunction. They define kidney manifestations as HTN, proteinuria ≥30 mg/dL or terminal complement activation. These criteria still need validation. HSCT-TMA diagnosis criteria are summarised in Table 3.

**Table 3:** Diagnostic criteria for Hematopoietic Stem Cell Transplantation (HSCT) associated Thrombotic Microangiopathy. Abbreviations: BMT-CTN – Bone Marrow Transplant Clinical Trials Network; HTN – hypertension; IWG – International Working Group; NS – not specified. a – Evidence of microangiopathy detected on tissue biopsy or laboratory and clinical markers, that could be the presence of any schistocytes, elevated lactate dehydrogenase, decreased haptoglobin and evidence of kidney dysfunction. b – Thrombocytopenia is defined by platelet <50 × 10^9^/L or >50% decrease in platelet count from baseline. c – Kidney dysfunction defined as doubling of serum creatinine or 50% decrease in creatinine clearance from baseline. Adapted from [127].

Criterion		BMT-CTN [123]	IWG [124]	Cho et al. [125]	Jodele et al. [126]
Schistocytes		>2 per high power field	>4%	>2 per high power field	Evidence of microangiopathy ^a^ (schistocytes or tissue biopsy)
Haemolysis	RBC	NS	Decreased hemoglobin or increased RBC transfusion	Decreased hemoglobin	Decreased hemoglobin or increased RBC transfusion
	Lactate dehydrogenase	Increased	Increased	Increased	Increased
	Haptoglobin	NS	Decreased	Decreased	Decreased
	Direct Coombs test	Negative	NS	Negative	NS
Thrombocytopenia ^b^		NS	Present	Present	Present
Kidney dysfunction ^c^		Present	NS	NS	Proteinuria >30 mg/dL or HTN
Neurologic features		Present	NS	NS	NS
Coagulation studies		Normal	Normal	Normal	NS
Terminal complement activation		NS	NS	NS	Elevated plasma concentration of sC5b-9.

### Treatment

The first line therapy consists of supportive measures, that may include discontinuing or reducing doses of CNI and mTORI, treatment of co-existing conditions (e.g., infections and GVHD), and tight control of HTN. Other nephrotoxins should be avoided any time close to the conditioning regimen [88], and platelet and RBC transfusion could be indicated [8].

According to several authors, successful prevention and treatment of GVHD is essential. Therefore, discontinuation of both CNI and mTORI may be controversial, with no evidence supporting it. However, dose reduction or replacement of one of them by other agents may be beneficial. There are successful reports of other strategies in GVHD, such as mycophenolate mofetil, corticosteroids and basiliximab (IL2 receptor antagonist) [88,92,97,99]. HSCT-TMA could also be minimized by protecting kidneys from TBI, e.g. fractionating irradiation over several days [128].

Several studies evaluated plasmapheresis efficacy in this setting, with controversial evidence. Some were not controlled, presenting numerous biases, such as different disease severity, heterogenous outcome evaluation, and the concomitant use of rituximab and defibrotide. The disappointing results may be explained by the fact that ADAMTS13 deficiency is not involved in the endothelial injury in this setting [90,91,99,129]. Nevertheless, plasmapheresis maintains its role in selected cases, because it can remove autoantibodies and complement activations products [130]. Retrospective studies showed at least partial benefit if it was started in early disease stages, with a higher overall survival [131], and improvement of hematologic manifestations [129]. No study, however, showed effectiveness in preventing CKD. Although not a cure, plasmapheresis still may be a temporizing measure, while evaluating for a more adequate strategy.

Several observational studies and case series reported other treatment options, all of which require further study. Defibrotide is a fibrinolytic, anti-thrombotic, anti-inflammatory, and thrombolytic agent, that could protect against endothelial damage by inhibition of TNFα (supported by in vitro studies) [132], [133], [134]. This drug protects endothelium from CNI and mTORI actions and has been used to prevent GVHD [134], [135], [136]. Two retrospective studies reported a 55% response rate with defibrotide in patients with grade 3 and 4 HSCT-TMA, without significant adverse effects [132,133]. Small retrospective studies reported improved outcomes with rituximab [137]. According to an analysis of several reports, approximately 80% of patients treated with rituximab (12 out of 15) showed response without significant toxicity [8]. Despite the encouraging results, studies on defibrotide and rituximab lack substantial numbers to validate this evidence [136].

While eculizumab has emerged as one of the most promising treatments for other TMAs, such as aHUS, its efficacy in HSCT-TMA is arguably modest. Retrospective studies that showed superior results when compared to plasmapheresis were based on small samples [100], [101], [102], [103], [104]. Worse outcomes were described in patients with higher sC5b-9, with a lower likelihood to respond to treatment [103]. More recently, Jodele et al. [103] showed a 50% rate of complete response to eculizumab, with severe disease being less responsive, which could suggest the existence of other targets of endothelial injury pathways. Besides, eculizumab comes with several challenges, namely the cost, timing of initiation, patient selection, dosing and duration of therapy [93,102]. The moderate therapeutic success achieved with eculizumab has led to investigation of other targeted therapies that act on various stages of the complement system. Some studies, still on phase II/III, already show encouraging results [93].

### Prognosis

In general, HSCT-TMA has a poor prognosis. The high mortality rate results from both increased morbidity and unsatisfying directed therapy [88,91,95,134,135].

The complexity of these patients makes them susceptible to several nephrotoxic insults. Regardless of the cause, requirement for kidney replacement therapy is always associated with worse prognosis [99].

Despite the arising number of available drugs in recent years, post-HSCT kidney dysfunction remains a significant complication [138]. A retrospective study showed that patients who recover from HSCT-TMA preserve only 40% of initial kidney function [99]. A more recent study revealed a cumulative incidence of severe CKD (requiring haemodialysis) of 33% [129].

Notwithstanding, recent studies have reported more promising survival rates [8,129].

## Tumor-induced TMA

Tumor-induced TMA (TiTMA) is a relatively rare paraneoplastic syndrome, with little incidence data. A retrospective French study analysed primary and secondary TMAs, reporting a TiTMA incidence of 19% [139]. Most patients had already been diagnosed with cancer, although it can occasionally be a presenting sign of malignancy [89,140]. Unlike other paraneoplastic syndromes, there is a high number of cases of TiTMA that occur with cancer recurrence, probably due to changes in tumor cells properties as a consequence of chemotherapy [141].

TiTMA is a diagnosis of exclusion, because many conditions related with cancer may trigger TMA, such as sepsis, chemotherapy and other drugs [89,139,142]. Review of the blood film and early bone marrow biopsy may accelerate the diagnosis of the underlying malignancy [7,14].

Most cases of TiTMA are associated with unspecific symptoms, with classical clinical and laboratory patterns present in only a minority of cases [141]. When TMA is the first manifestation of an occult malignancy, patients could present an abrupt onset of anemia and thrombocytopenia. In cancer patients presenting with unexplained anemia and thrombocytopenia, it is essential to exclude BM metastasis. Kidney dysfunction is less common when compared with HSCT-TMA or cancer DiTMA [89,143].

If patients present atypical clinical features of TMA or fail to respond to treatments for idiopathic TMA, some authors recommend searching for systemic malignancy, after excluding other secondary causes [14,141,143,144].

In the following pages, we describe the specificities of TMA in solid and hematologic tumours.

### Solid tumours

The solid tumours most commonly associated with TMA are mucinous adenocarcinomas, namely gastric, breast, pancreas, prostate and lung. Usually, patients present a metastatic cancer when TMA is diagnosed [34,141,143].

#### Mechanisms

Several mechanisms for TMA in solid tumours have been proposed, although its pathogenesis is not completely clarified yet [141].

Measuring ADAMTS13 in this context may not be useful, as it could range from undetectable to normal levels in metastatic cancer and its value is not related with TMA [89,141,143]. In fact, the majority of the patients present an adequate activity level of this enzyme, suggesting that ADAMTS13 dysfunction is not prominent in TiTMA [89,143].

Several case series reported a high prevalence of BM metastasis in patients with TiTMA. In those cases, angiogenesis inside the BM is probably increased, a condition associated with aggressive tumor growth and secondary myelofibrosis. These mechanisms would lead to endothelial injury, beginning the cascade that leads to TMA [143]. However, BM metastasis is not searched for in all patients and in a substantial number of published cases in which the BM was studied there was no evidence of metastasis [141].

Adenocarcinoma is the histologic type most related to TMA. Some authors defend that mucin production may exert a direct injury in the tumoral endothelial cells. Microvascular tumor emboli, procoagulants produced by tumor cells and impaired fibrinolysis have also been implicated [34,89,139,141,143].

#### Clinical manifestations

A cross-sectional multicentre study [143] showed that patients with TiTMA, when compared with those with idiopathic TMA, had a longer duration of symptoms, including some atypical ones, such as bone pain and wasting. Upon diagnosis, thrombocytopenia and kidney injury were less severe.

In general, there is an increased risk for thrombotic complications. Pulmonary TMA is a rare condition, but more common in TiTMA patients, being characterized by intimal proliferation of the pulmonary arterioles with presence of tumor emboli [141,142].

#### Treatment and prognosis

Treatment of the underlying cancer is the cornerstone of therapy. Several case reports describe a fast hematologic response, sometimes achieved after one cycle of chemotherapy. Patients who initiate early chemotherapy have a better prognosis, with a considerably higher survival rate and a good quality of life. Plasmapheresis could be useful if antibody-mediated TMA is suspected [10,142].

In general, TMA related to solid tumours has a very poor prognosis, probably because most patients present metastatic disease. Some series report a mortality rate of almost 50% [89,142,143].

### Hematologic tumours

Most works about haemolytic anemia in the context of hematologic tumor report all kinds of causes, with only a few studies focusing on TMA. Besides, the majority includes both solid and hematologic tumours, with a broad incidence rate for the latter – 8% to 50% of TiTMA [34,139,141,142]. The most common hematologic tumours behind TiTMA are Hodgkin lymphoma, aggressive Non-Hodgkin lymphomas, acute leukaemia and multiple myeloma [34,141,144].

In a recent case series, the prevalence of monoclonal gammopathy in a TMA population was five-folder higher than expected (21% versus 4,2%, in patients 50 and older), which may indicate an association between those entities [147].

#### Mechanisms

The mechanisms for hematologic TiTMA are not well recognized. Several heterogeneous factors are likely to contribute, given the broad spectrum of hematologic tumours involved.

A few cases of TMA related with lymphoma and myeloma had detectable antibodies against ADAMTS13, which disappeared once achieving malignancy remission [127,141].

Monoclonal immunoglobulins (MIg), in particular, may act as TMA triggers or facilitators, possibly through both direct and indirect mechanisms. Direct injury to endothelial cells makes them more susceptible to TMA after another insult [147]. On the other hand, indirect mechanisms rely on inhibiting proteins that regulate thrombosis, such as ADAMTS13, VWF, platelet membrane glycoprotein 1b and complement factor H. In this context, MIg act as an autoantibody. When the target is complement factor H, the paraprotein overactivates the alternative complement pathway, leading to C3 glomerulopathy or aHUS [147], [148], [149].

Clearly, further studies are required to determine the mechanisms of TMA, given both the rarity of this situation and the common presence of confounding factors.

#### Clinical manifestations

A thorough evaluation is essential, in order to identify atypical signs and symptoms that allow the recognition of an indolent hematologic disease. For instance, although increased serum LDH is characteristic of TMA, extreme elevations are atypical and may suggest tumor lysis syndrome [144].

In a series of 9 cases of TMA related with monoclonal gammopathies [149], all the patients presented with a kidney-limited TMA, with no confounding factors on presentation. It has to be noted, however, that genetic mutations on complement factors and ADAMTS13 activity were not evaluated in all patients. By treating the underlying gammopathy, the TMA was reversed, which suggests a strong association between these entities.

#### Treatment and prognosis

Just like in any secondary TMA, treatment of the underlying disease is mandatory [141,147]. There are case series reporting failure to respond to eculizumab [147].

When antibody-mediated TMA is suspected, a trial with plasmapheresis could be useful. However, some cases only respond with complete malignancy remission [141].

There is no evidence on plasmapheresis for treatment of monoclonal gammopathy in this setting.

### Disseminated intravascular coagulation

Disseminated intravascular coagulation (DIC), a condition closely related to cancer, must be considered in the differential diagnosis of MAHA and thrombocytopenia in these patients. It is a potentially life-threatening condition, defined as a systemic process that can lead to hemorrhage and thrombosis, with organ injury. The pathogenesis consists of an impaired coagulation, in association with a microvascular thrombosis due to neutrophil activation and endothelial damage [145]. Several authors consider DIC as a TMA, because it could manifest with MAHA. However, characteristic features of other TMA are uncommon in DIC, such as severe thrombocytopenia, normal coagulation studies and platelet-rich microthrombi (without significant fibrin) [142,146]. DIC pathogenesis also suggests that a procoagulant exposure may initiate the disease [145], although it shares many potential triggers with TMA [146]. Scoring systems were developed to improve diagnosis accuracy of DIC, such as the ISTH (International Society of Thrombosis and Hemostasis) score, with a sensitivity and specificity above 90% [7,146].

## Conclusions

Despite its rarity, TMA in Oncology is a potentially fatal condition whose varied pathophysiological features remain vastly unknown. A high grade of suspicion is therefore crucial for a prompt recognition and treatment.

The recent trend of rapid increase in the approval of new drugs brings challenges to the management of TMA. In fact, introducing new targeted therapies may lead to nephrotoxicity by unexpected mechanisms. ADAMTS13 activity could help clinicians to decide if the initial treatment should be plasmapheresis or complement inhibition [83].

In what concerns HSCT-induced TMA, the high number of confounding factors have made it difficult to understand its underlying mechanisms. tumor-induced TMA, in contrast, was recognized decades ago in the context of solid tumours; in the last few years, comprehension of TMA in onco-hematological patients was significantly improved.

Nevertheless, for any assumed trigger, the current scientific literature points out complement cascade mutations as potential TMA facilitators.

At the present moment, there is no solid evidence supporting any particular therapeutic approaches. Randomized controlled trials, an especially difficult achievement in a field like this, would be the only way to make it possible to recommend a specific therapy.

## CRediT authorship contribution statement

**Patrícia Valério:** Conceptualization, Methodology, Investigation, Writing – original draft, Writing – review & editing. **João Pedro Barreto:** Conceptualization, Methodology, Writing – review & editing. **Hugo Ferreira:** Supervision, Writing – review & editing. **Teresa Chuva:** Supervision, Writing – review & editing. **Ana Paiva:** Supervision, Writing – review & editing. **José Maximino Costa:** Conceptualization, Methodology, Investigation, Supervision, Writing – review & editing.

## Declaration of Competing Interest

The authors declare that they have no known competing financial interests or personal relationships that could have appeared to influence the work reported in this paper.

## References

[1] George J.N., Nester C.M. (2014). Syndromes of thrombotic microangiopathy. N. Engl. J. Med..

[2] Moschcowitz E. (1924). Hyaline thrombosis of the terminal arterioles and capillaries: a hitherto undescribed disease. Proc. N Y Pathol. Soc..

[3] Singer K., Bornstein F.P., Wile S.A. (1947). Thrombotic thrombocytopenic purpura; hemorrhagic diathesis with generalized platelet thromboses. Blood.

[4] Azevedo A., Faria B., Teixeira C., Carvalho F., Neto G., Santos J. (2018). Portuguese consensus document statement in diagnostic and management of atypical hemolytic uremic syndrome. Port J. Nephrol. Hypert..

[5] Weitz I.C. (2019). Thrombotic Microangiopathy in cancer. Semin. Thromb Hemost..

[6] Izzedine H., Perazella M.A. (2015). Thrombotic microangiopathy, cancer, and cancer drugs. Am. J. Kidney Dis..

[7] Thomas M.R., Scully M. (2019). Microangiopathy in cancer: causes, consequences, and management. Cancer Treat Res..

[8] Wanchoo R., Bayer R.L., Bassil C., Jhaveri K.D. (2018). Emerging concepts in hematopoietic stem cell transplantation-associated renal thrombotic microangiopathy and prospects for new treatments. Am. J. Kidney Dis..

[9] Goodship T.H., Cook H.T., Fakhouri F., Fervenza F.C., Fremeaux-Bacchi V., Kavanagh D. (2017). Atypical hemolytic uremic syndrome and C3 glomerulopathy: conclusions from a "kidney disease: improving global outcomes" (kdigo) controversies conference. Kidney Int..

[10] Padmanabhan A., Connelly-Smith L., Aqui N., Balogun R.A., Klingel R., Meyer E. (2019). Guidelines on the use of therapeutic apheresis in clinical practice - evidence-based approach from the writing committee of the american society for apheresis: the eighth special issue. J. Clin. Apher..

[11] Ritchie G.E., Fernando M., Goldstein D. (2017). Rituximab to treat gemcitabine-induced hemolytic-uremic syndrome (Hus) in pancreatic adenocarcinoma: a case series and literature review. Cancer Chemother. Pharmacol..

[12] Turner J.L., Reardon J., Bekaii-Saab T., Cataland S.R., Arango M.J. (2016). Gemcitabine-associated thrombotic microangiopathy: response to complement inhibition and reinitiation of gemcitabine. Clin. Colorectal Cancer.

[13] Walter R.B., Joerger M., Pestalozzi B.C. (2002). Gemcitabine-associated hemolytic-uremic syndrome. Am. J. Kidney Dis..

[14] Morton J.M., George J.N. (2016). Microangiopathic hemolytic anemia and thrombocytopenia in patients with cancer. J. Oncol. Pract..

[15] Chatzikonstantinou T., Gavriilaki M., Anagnostopoulos A., Gavriilaki E. (2020). An update in drug-induced thrombotic microangiopathy. Front. Med. (Lausanne).

[16] Institute NC. (2017). Common Terminology Criteria for Adverse Events (Ctcae) - Version 5.0.

[17] Mitomycin-C Pulmonary Toxicity [Internet]. (Accessed July 16, 2020). 2020. Available from: https://www.uptodate.com/contents/mitomycin-c-pulmonary-toxicity?search=Mitomycin%20C&source=search_result&selectedTitle=2~106&usage_type=default&display_rank=1.

[18] Liu K., Mittelman A., Sproul E.E., Elias E.G. (1971). Renal toxicity in man treated with mitomycin C. Cancer.

[19] Valavaara R., Nordman E. (1985). Renal complications of mitomycin c therapy with special reference to the total dose. Cancer.

[20] Dlott J.S., Danielson C.F., Blue-Hnidy D.E., McCarthy L.J. (2004). Drug-induced thrombotic thrombocytopenic purpura/hemolytic uremic syndrome: a concise review. Ther. Apher. Dial.

[21] Verweij J., Van der Burg M.E., Pinedo H.M. (1987). Mitomycin C-induced hemolytic uremic syndrome. six case reports and review of the literature on renal, pulmonary and cardiac side effects of the drug. Radiother. Oncol..

[22] Onitilo A.A., Engel J.M., Clouse L.H., Gerndt K.M. (2009). Successful treatment of mitomycin-induced thrombotic thrombocytopenic purpura with rituximab. J. Vasc. Interv. Radiol..

[23] Lesesne J.B., N. R., Erickson B., B E., Korec S., Sisk R. (1989). Cancer-associated hemolytic-uremic syndrome: analysis of 85 cases from a national registry. J. Clin. Oncol..

[24] Reese J.A., BD W., Curtis B.R., Terrell S.R., Vesely S.K., Aster R.H. (2015). Drug-induced thrombotic microangiopathy: experience of the oklahoma registry and the bloodcenter of Wisconsin. Am. J. Hematol..

[25] Sheldon R., Slaughter D. (1986). A syndrome of microangiopathic hemolytic anemia, renal impairment, and pulmonary edema in chemotherapy-treated patients with adenocarcinoma. Cancer.

[26] Zeller G., E. W., Schwarting A. (2003). Mitomycin-induced hemolytic-uremic syndrome. Dtsch Med. Wochenschr.

[27] Shah G., Yamin H., Smith H. (2013). Mitomycin-C-induced Ttp/hus treated successfully with rituximab: case report and review of the literature. Case Rep. Hematol..

[28] Faguer S., Huart A., Fremeaux-Bacchi V., Ribes D., Chauveau D. (2013). Eculizumab and drug-induced haemolytic-uraemic syndrome. Clin. Kidney J..

[29] Hausberg M., Felten H., Pfeffer S. (2019). Treatment of chemotherapy-induced thrombotic microangiopathy with eculizumab in a patient with metastatic breast cancer. Case Rep. Oncol..

[30] Izzedine H., Isnard-Bagnis C., Launay-Vacher V., Mercadal L., Tostivint I., Rixe O. (2006). Gemcitabine-induced thrombotic microangiopathy: a systematic review. Nephrol. Dial Trans..

[31] Glezerman I.G., G. K.M., Miller V., V M., Seshan S., Flombaum C.D. (2009). Gemcitabine nephrotoxicity and hemolytic uremic syndrome: report of 29 cases from a single institution. Clin. Nephrol..

[32] Saif M.W., Xyla V., Makrilia N., Bliziotis I., Syrigos K. (2009). Thrombotic microangiopathy associated with gemcitabine: rare but real. Expert Opin Drug Saf..

[33] Fung M.C., Storniolo A.M., Nguyen B., Arning M., Brookfield W., Vigil J. (1999). A review of hemolytic uremic syndrome in patients treated with gemcitabine therapy. Cancer.

[34] Kheder El-Fekih R., Deltombe C., Izzedine H. (2017). Thrombotic microangiopathy and cancer. Nephrol. Ther..

[35] López Rubio M.E., Rodado Martinez R., Illescas M.L., Mateo Bosch E., Martinez Diaz M., de la Vara Inesta L. (2017). Gemcitabine-induced hemolytic-uremic syndrome treated with eculizumab or plasmapheresis: two case reports. Clin. Nephrol..

[36] Bharthuar A., Egloff L., Becker J., George M., Lohr J.W., Deeb G. (2009). Rituximab-based therapy for gemcitabine-induced hemolytic uremic syndrome in a patient with metastatic pancreatic adenocarcinoma: a case report. Cancer Chemother Pharmacol..

[37] Gourley B.L., Mesa H., Gupta P. (2010). Rapid and complete resolution of chemotherapy-induced thrombotic thrombocytopenic purpura/hemolytic uremic syndrome (ttp/hus) with rituximab. Cancer Chemother. Pharmacol..

[38] Al Ustwani O., Lohr J., Dy G., Levea C., Connolly G., Arora P. (2014). Eculizumab therapy for gemcitabine induced hemolytic uremic syndrome: case series and concise review. J. Gastrointest Oncol..

[39] Krishnappa V., Gupta M., Shah H., Das A., Tanphaichitr N., Novak R. (2018). The use of eculizumab in gemcitabine induced thrombotic microangiopathy. BMC Nephrol..

[40] Cisplatin Nephrotoxicity [Internet]. (Accessed July 16, 2020). 2020. Available from: https://www.uptodate.com/contents/cisplatin-nephrotoxicity?search=Cisplatin%20nephrotoxicity&source=search_result&selectedTitle=1~150&usage_type=default&display_rank=1.

[41] Gilbert R.D., Stanley L.K., Fowler D.J., Angus E.M., Hardy S.A., Goodship T.H. (2013). Cisplatin-induced haemolytic uraemic syndrome associated with a novel intronic mutation of Cd46 treated with eculizumab. Clin. Kidney J..

[42] Gardner G., Mesler D., Gitelman H.J. (1989). Hemolytic uremic syndrome following cisplatin, bleomycin, and vincristine chemotherapy: a report of a case and a review of the literature. Ren. Fail.

[43] Jackson A., Rose B.D., Graff L.G., Jacobs J.B., Schwartz J.H., Strauss G.M. (1984). Thrombotic Microangiopathy and renal failure associated with antineoplastic chemotherapy. Ann. Intern Med..

[44] Dieckmann K.P., Struss W.J., Budde U. (2011). Evidence for acute vascular toxicity of cisplatin-based chemotherapy in patients with germ cell tumour. Anticancer Res..

[45] Canpolat C., Pearson P., Jaffe N. (1994). Cisplatin-associated hemolytic uremic syndrome. Cancer.

[46] Gradishar W.J., Vokes E.E., Ni K., Panje W.R. (1990). Chemotherapy-related hemolytic-uremic syndrome after the treatment of head and neck cancer. a case report. Cancer.

[47] Muto J., Kishimoto H., Kaizuka Y., Kinjo M., Higashi H., Kishihara F. (2017). Thrombotic microangiopathy following chemotherapy with S-1 and cisplatin in a patient with gastric cancer: a case report. In Vivo (Brooklyn).

[48] Palmisano J., Agraharkar M., Kaplan A.A. (1998). Successful treatment of cisplatin-induced hemolytic uremic syndrome with therapeutic plasma exchange. Am. J. Kidney Dis..

[49] Niu J., Mims M.P. (2012). Oxaliplatin-induced thrombotic thrombocytopenic purpura: case report and literature review. J. Clin. Oncol.

[50] Zanchelli F., Tampieri E., Gozzetti F., Monti M., Martelli D., Graziani R. (2017). Atypical Hemolytic uremic syndrome related to oxalyplatin cancer chemotherapy responsive to eculizumab. G Ital Nefrol..

[51] Iams W., Beckermann K.E., Neff A.T., Mayer I.A., Abramson V.G. (2013). Thrombotic microangiopathy during docetaxel, trastuzumab, and carboplatin chemotherapy for early-stage her2+ breast cancer: a case report. Med. Oncol..

[52] Gross M., Hiesse C., Fau - Kriaa F., Kriaa F., Fau - Goldwasser F., Goldwasser F. (1999). Severe hemolytic uremic syndrome in an advanced ovarian cancer patient treated with carboplatin and gemcitabine. Anticancer Drugs.

[53] Walker R.W., Rosenblum, Kempin S.J., Christian M.C. (1989). Carboplatin-associated thrombotic microangiopathic hemolytic anemia. Cancer.

[54] Shavit L., Lifschitz M.D., Gabizon A., Kwa M., Muggia F., Slotki I. (2014). Pegylated liposomal doxorubicin and renal thrombotic microangiopathy: an under-recognized complication of prolonged treatment for ovarian cancer. Kidney Int..

[55] Gabizon A., Shmeeda H., Barenholz Y. (2003). Pharmacokinetics of pegylated liposomal doxorubicin: review of animal and human studies. Clin. Pharmacokinet.

[56] Kwa M., Baumgartner R.F., Shavit L., Barash I., Michael J., Puzanov I. (2012). Is renal thrombotic angiopathy an emerging problem in the treatment of ovarian cancer recurrences?. Oncologist.

[57] Weitz I.C., Deloughery T. (2018). Effective treatment of chemotherapy induced atypical haemolytic uraemic syndrome: a case series of 7 treated patients. Br. J. Haematol..

[58] Siau K., Varughese M. (2010). Thrombotic microangiopathy following docetaxel and trastuzumab chemotherapy: a case report. Med. Oncol..

[59] Shrestha A., Khosla P., Wei Y. (2011). Docetaxel-induced thrombotic thrombocytopenic purpura/hemolytic uremic syndrome-related complex in a patient with metastatic prostate cancer?. Am. J. Ther..

[60] Strati P., Nasr S.H., Leung N., Hanson C.A., Chaffee K.G., Schwager S.M. (2015). Renal complications in chronic lymphocytic leukemia and monoclonal b-cell lymphocytosis: the mayo clinic experience. Haematologica.

[61] Jhaveri K.D., Wanchoo R., Sakhiya V., Ross D.W., Fishbane S. (2016). Adverse renal effects of novel molecular oncologic targeted therapies: a narrative review. Kidney Int. Rep..

[62] Izzedine H., Escudier B., Lhomme C., Pautier P., Rouvier P., Gueutin V. (2014). Kidney diseases associated with anti-vascular endothelial growth factor (vegf): an 8-year observational study at a single center. Medicine (Baltimore).

[63] Eremina V., Jefferson J.A., Kowalewska J., Hochster H., Haas M., Weisstuch J. (2008). Vegf inhibition and renal thrombotic microangiopathy. N. Engl. J. Med..

[64] Vigneau C., Lorcy N., Dolley-Hitze T., Jouan F., Arlot-Bonnemains Y., Laguerre B. (2014). All anti-vascular endothelial growth factor drugs can induce 'pre-eclampsia-like syndrome': a rare study. Nephrol. Dial Trans..

[65] Cross S.N., Ratner E., Rutherford T.J., Schwartz P.E., Norwitz E.R. (2012). Bevacizumab-mediated interference with Vegf signaling is sufficient to induce a preeclampsia-like syndrome in nonpregnant women. Rev. Obstet Gynecol..

[66] Moghaddas Sani H., Zununi Vahed S., Ardalan M. (2019). Preeclampsia: a close look at renal dysfunction. Biomed Pharmacother..

[67] Bollee G., Patey N., Cazajous G., Robert C., Goujon J.M., Fakhouri F. (2009). Thrombotic microangiopathy secondary to Vegf pathway inhibition by sunitinib. Nephrol. Dial Trans..

[68] Vakiti A., Singh D., Pilla R., Alhaj-Moustafa M., Fitzpatrick K.W. (2019). Bevacizumab-induced atypical hemolytic uremic syndrome and treatment with eculizumab. J. Oncol. Pharm. Pract..

[69] Blake-Haskins J.A., Lechleider R.J., Kreitman R.J. (2011). Thrombotic microangiopathy with targeted cancer agents. Clin. Cancer Res..

[70] Patel T.V., Jeffrey A., Morgan J.A., Demetri G.D., George S., Maki R.G. (2008). A preeclampsia-like syndrome characterized by reversible hypertension and proteinuria induced by the multitargeted kinase inhibitors sunitinib and sorafenib. J. Natl. Cancer Inst..

[71] Kapiteijn E., Brand A., Kroep J., Gelderblom H. (2007). Sunitinib induced hypertension, thrombotic microangiopathy and reversible posterior leukencephalopathy syndrome. Ann. Oncol..

[72] Ojeda-Uribe M., Merieau S., Guillon M., Aujoulat O., Hinschberger O., Eisenmann J.C. (2016). Secondary thrombotic microangiopathy in two patients with philadelphia-positive hematological malignancies treated with imatinib mesylate. J. Oncol. Pharm. Pract..

[73] Al Aly Z., Philoctete Ashley J.M., Gellens M.E., Gonzalez E.A. (2005). Thrombotic thrombocytopenic purpura in a patient treated with imatinib mesylate: true association or mere coincidence?. Am. J. Kidney Dis..

[74] Feldman D.R., Baum M.S., Ginsberg S.M., Hassoun H., Flombaum C.D. (2009). Phase I trial of bevacizumab plus escalated doses of sunitinib in patients with metastatic renal cell carcinoma. J. Clin. Oncol..

[75] Yui J.C., Van Keer J., Weiss B.M., Waxman A.J., Palmer M.B., D'Agati V.D. (2016). Proteasome inhibitor associated thrombotic microangiopathy. Am. J. Hematol..

[76] Lodhi A., Kumar A., Saqlain M.U., Suneja M. (2015). Thrombotic microangiopathy associated with proteasome inhibitors. Clin. Kidney J..

[77] Field-Smith A., Morgan G.J., Davies F.E. (2006). Bortezomib (velcadetrade mark) in the treatment of multiple myeloma. Ther. Clin. Risk Manag..

[78] Wanchoo R., Khan S., Kolitz J.E., Jhaveri K.D. (2015). Carfilzomib-related acute kidney injury may be prevented by N-acetyl-l-cysteine. J. Oncol. Pharm. Pract..

[79] Moore H., Romeril K. (2011). Multiple myeloma presenting with a fever of unknown origin and development of thrombotic thrombocytopenic purpura post-bortezomib. Intern Med. J..

[80] Saleem R., Reese J.A., George J.N. (2018). Drug-induced thrombotic microangiopathy: an updated systematic review, 2014–2018. Am. J. Hematol..

[81] Atallah-Yunes S.A., Soe M.H. (2018). Drug-induced thrombotic microangiopathy due to cumulative toxicity of ixazomib. Case Rep. Hematol..

[82] Yui J.C., Dispenzieri A., Leung N. (2017). Ixazomib-induced thrombotic microangiopathy. Am. J. Hematol..

[83] Gosain R., Gill A., Fuqua J., Volz L.H., Kessans Knable M.R., Bycroft R. (2017). Gemcitabine and carfilzomib induced thrombotic microangiopathy: eculizumab as a life-saving treatment. Clin. Case Rep..

[84] Portuguese A.J., Lipe B. (2018). Carfilzomib-induced Ahus responds to early eculizumab and may be associated with heterozygous Cfhr3-Cfhr1 deletion. Blood Adv..

[85] Cortazar F.B., Marrone K.A., Troxell M.L., Ralto K.M., Hoenig M.P., Brahmer J.R. (2016). Clinicopathological features of acute kidney injury associated with immune checkpoint inhibitors. Kidney Int..

[86] Yamada R., Okawa T., Matsuo K., Suzuki M., Mori N., Mori K. (2019). Renal-limited thrombotic microangiopathy after switching from bevacizumab to ramucirumab: a case report. BMC Nephrol..

[87] Koizumi M., Takahashi M., Murata M., Kikuchi Y., Seta K., Yahata K. (2017). Thrombotic microangiopathy associated with cetuximab, an epidermal growth factor receptor inhibitor. Clin. Nephrol..

[88] Gavriilaki E., Sakellari I., Batsis I., Mallouri D., Bousiou Z., Vardi A. (2018). Transplant-associated thrombotic microangiopathy: incidence, prognostic factors, morbidity, and mortality in allogeneic hematopoietic cell transplantation. Clin. Trans..

[89] Qu L., Kiss J.E. (2005). Thrombotic microangiopathy in transplantation and malignancy. Semin. Thromb Hemost..

[90] Iacopino P., Pucci G., Arcese W., Bosi A., Falda M., Locatelli F. (1999). Severe thrombotic microangiopathy: an infrequent complication of bone marrow transplantation. gruppo italiano trapianto midollo osseo (Gitmo). Bone Marrow Trans..

[91] George J.N., Li X., McMinn J.R., Terrell D.R., Vesely S.K., Selby G.B. (2004). Thrombotic thrombocytopenic purpura-hemolytic uremic syndrome following allogeneic Hpc transplantation: a diagnostic dilemma. Transfusion.

[92] Jodele S. (2018). Complement in pathophysiology and treatment of transplant-associated thrombotic microangiopathies. Semin. Hematol..

[93] Gavriilaki E., Anagnostopoulos A., Mastellos D.C. (2019). Complement in thrombotic microangiopathies: unraveling ariadne's thread into the labyrinth of complement therapeutics. Front. Immunol..

[94] Labrador J., Lopez-Corral L., Lopez-Godino O., Vazquez L., Cabrero-Calvo M., Perez-Lopez R. (2014). Risk factors for thrombotic microangiopathy in allogeneic hematopoietic stem cell recipients receiving Gvhd prophylaxis with tacrolimus plus Mtx or sirolimus. Bone Marrow Trans..

[95] Shayani S., Palmer J., Stiller T., Liu X., Thomas S.H., Khuu T. (2013). Thrombotic microangiopathy associated with sirolimus level after allogeneic hematopoietic cell transplantation with tacrolimus/sirolimus-based graft-versus-host disease prophylaxis. Biol Blood Marrow Trans..

[96] Willems E., Baron F., Seidel L., Frere P., Fillet G., Beguin Y. (2010). Comparison of thrombotic microangiopathy after allogeneic hematopoietic cell transplantation with high-dose or nonmyeloablative conditioning. Bone Marrow Trans..

[97] Changsirikulchai S., Myerson D., Guthrie K.A., McDonald G.B., Alpers C.E., Hingorani S.R. (2009). Renal thrombotic microangiopathy after hematopoietic cell transplant: role of Gvhd in pathogenesis. Clin. J. Am. Soc. Nephrol..

[98] Roy V., Rizvi M.A., Vesely S.K., George J.N. (2001). Thrombotic thrombocytopenic purpura-like syndromes following bone marrow transplantation: an analysis of associated conditions and clinical outcomes. Bone Marrow Trans..

[99] Laskin B.L., Goebel J., Davies S.M., Jodele S. (2011). Small vessels, big trouble in the kidneys and beyond: hematopoietic stem cell transplantation-associated thrombotic microangiopathy. Blood.

[100] de Fontbrune F.S., Galambrun C., Sirvent A., Huynh A., Faguer S., Nguyen S. (2015). Use of eculizumab in patients with allogeneic stem cell transplant-associated thrombotic microangiopathy: a study from the Sfgm-Tc. Transplantation.

[101] Peffault de Latour R., Xhaard A., Fremeaux-Bacchi V., Coppo P., Fischer A.M., Helley D. (2013). Successful use of eculizumab in a patient with post-transplant thrombotic microangiopathy. Br. J. Haematol..

[102] Jodele S., Fukuda T., Vinks A., Mizuno K., Laskin B.L., Goebel J. (2014). Eculizumab therapy in children with severe hematopoietic stem cell transplantation-associated thrombotic microangiopathy. Biol Blood Marrow Trans..

[103] Jodele S., Dandoy C.E., Lane A., Laskin B.L., Teusink-Cross A., Myers K.C. (2020). Complement blockade for Ta-Tma: lessons learned from a large pediatric cohort treated with eculizumab. Blood.

[104] Jodele S., Dandoy C.E., Myers K.C., El-Bietar J., Nelson A., Wallace G. (2016). New approaches in the diagnosis, pathophysiology, and treatment of pediatric hematopoietic stem cell transplantation-associated thrombotic microangiopathy. Transfus Apher. Sci..

[105] Jodele S., Davies S.M., Lane A., Khoury J., Dandoy C., Goebel J. (2014). Diagnostic and risk criteria for Hsct-associated thrombotic microangiopathy: a study in children and young adults. Blood.

[106] Gavriilaki E., Chrysanthopoulou A., Sakellari I., Batsis I., Mallouri D., Touloumenidou T. (2019). Linking complement activation, coagulation, and neutrophils in transplant-associated thrombotic microangiopathy. Thromb Haemost..

[107] Jodele S., Licht C., Goebel J., Dixon B.P., Zhang K., Sivakumaran T.A. (2013). Abnormalities in the alternative pathway of complement in children with hematopoietic stem cell transplant-associated thrombotic microangiopathy. Blood.

[108] Jodele S., Zhang K., Zou F., Laskin B., Dandoy C.E., Myers K.C. (2016). The genetic fingerprint of susceptibility for transplant-associated thrombotic microangiopathy. Blood.

[109] Gavriilaki E., Touloumenidou T., Sakellari I., Batsis I., Mallouri D., Psomopoulos F. (2020). Pretransplant genetic susceptibility: clinical relevance in transplant-associated thrombotic microangiopathy. Thromb Haemost.

[110] Laskin B.L., Maisel J., Goebel J., Yin H.J., Luo G., Khoury J.C. (2013). Renal Arteriolar C4d Deposition: a novel characteristic of hematopoietic stem cell transplantation-associated thrombotic microangiopathy. Transplantation.

[111] Biedermann B.C., Sahner S., Gregor M., Tsakiris D.A., Jeanneret C., Pober J.S. (2002). Endothelial injury mediated by cytotoxic t lymphocytes and loss of microvessels in chronic graft versus host disease. Lancet.

[112] Matsuda Y., Hara J., Osugi Y., Tokimasa S., Fujisaki H., Takai K. (2001). Serum levels of soluble adhesion molecules in stem cell transplantation-related complications. Bone Marrow Trans..

[113] Chan G.S., Lam M.F., Au W.Y., Chim S., Tse K.C., Lo S.H. (2008). Clinicopathologic analysis of renal biopsies after haematopoietic stem cell transplantation. Nephrology (Carlton).

[114] Perez-Simon J.A., Martino R., Parody R., Cabrero M., Lopez-Corral L., Valcarcel D. (2013). The combination of sirolimus plus tacrolimus improves outcome after reduced-intensity conditioning, unrelated donor hematopoietic stem cell transplantation compared with cyclosporine plus mycofenolate. Haematologica.

[115] Perkowska-Ptasinska A., Sulikowska-Rowinska A., Pazik J., Komuda-Leszek E., Durlik M. (2006). Thrombotic nephropathy and pulmonary hypertension following autologous bone marrow transplantation in a patient with acute lymphoblastic leukemia: case report. Trans. Proc..

[116] Schoettler M., Lehmann L., Li A., Ma C., Duncan C. (2019). Thrombotic microangiopathy following pediatric autologous hematopoietic cell transplantation: a report of significant end-organ dysfunction in eculizumab-treated survivors. Biol Blood Marrow Trans..

[117] Jodele S., Dandoy C.E., Myers K., Wallace G., Lane A., Teusink-Cross A. (2018). High-dose carboplatin/etoposide/melphalan increases risk of thrombotic microangiopathy and organ injury after autologous stem cell transplantation in patients with neuroblastoma. Bone Marrow Trans..

[118] Overview and Clinical Assessment of the Complement System [Internet]. (Accessed December 2, 2020). 2020. Available from: https://www.uptodate.com/contents/overview-and-clinical-assessment-of-the-complement-system?search=complement&source=search_result&selectedTitle=1~150&usage_type=default&display_rank=1.

[119] Horvath O., Kallay K., Csuka D., Mezo B., Sinkovits G., Kassa C. (2018). Early increase in complement terminal pathway activation marker Sc5b-9 is predictive for the development of thrombotic microangiopathy after stem cell transplantation. Biol Blood Marrow Trans..

[120] Qi J., Wang J., Chen J., Su J., Tang Y., Wu X. (2017). Plasma levels of complement activation fragments C3b and Sc5b-9 significantly increased in patients with thrombotic microangiopathy after allogeneic stem cell transplantation. Ann. Hematol..

[121] Sartain S., Shubert S., Wu M.F., Wang T., Martinez C. (2020). The alternative complement pathway activation product ba as a marker for transplant-associated thrombotic microangiopathy. Pediatr Blood Cancer.

[122] Laskin B.L., Goebel J., Davies S.M., Khoury J.C., Bleesing J.J., Mehta P.A. (2011). Early clinical indicators of transplant-associated thrombotic microangiopathy in pediatric neuroblastoma patients undergoing auto-Sct. Bone Marrow Transplant.

[123] Ho V.T., Cutler C., Carter S., Martin P., Adams R., Horowitz M. (2005). Blood and marrow transplant clinical trials network toxicity committee consensus summary: thrombotic microangiopathy after hematopoietic stem cell transplantation. Biol Blood Marrow Trans..

[124] Ruutu T., G. B., Benjamin R.J., Clark R.E., George J.N., Gratwohl A. (2007). Diagnostic Criteria for Hematopoietic Stem Cell Transplant-Associated Microangiopathy: Results of a Consensus Process By an International Working Group.

[125] Cho B.S., Yahng S.A., Lee S.E., Eom K.S., Kim Y.J., Kim H.J. (2010). Validation of recently proposed consensus criteria for thrombotic microangiopathy after allogeneic hematopoietic stem-cell transplantation. Transplantation.

[126] Jodele S., Laskin B.L., Dandoy C.E., Myers K.C., El-Bietar J., Davies S.M. (2015). A new paradigm: diagnosis and management of Hsct-associated thrombotic microangiopathy as multi-system endothelial injury. Blood Rev..

[127] Elsallabi O., Bhatt V.R., Dhakal P., Foster K.W., Tendulkar K.K. (2016). Hematopoietic stem cell transplant-associated thrombotic microangiopathy. Clin. Appl. Thromb Hemost..

[128] Leblond V., Sutton L., Jacquiaud C., tem C., Sadoun R., Jaudon M.C. (1995). Evaluation of renal function in 60 long-term survivors of bone marrow transplantation. J. Am. Soc. Nephrol..

[129] Sartain S., Shubert S., Wu M.F., Srivaths P., Teruya J., Krance R. (2019). Therapeutic plasma exchange does not improve renal function in hematopoietic stem cell transplantation-associated thrombotic microangiopathy: an institutional experience. Biol Blood Marrow Trans..

[130] Worel N., Greinix H.T., Leitner G., Mitterbauer M., Rabitsch W., Rosenmayr A. (2007). Abo-incompatible allogeneic hematopoietic stem cell transplantation following reduced-intensity conditioning: close association with transplant-associated microangiopathy. Transfus. Apher. Sci..

[131] Jodele S., Laskin B.L., Goebel J., Khoury J.C., Pinkard S.L., Carey P.M. (2013). Does early initiation of therapeutic plasma exchange improve outcome in pediatric stem cell transplant-associated thrombotic microangiopathy?. Transfusion.

[132] Corti P., Uderzo C., Tagliabue A., Della Volpe A., Annaloro C., Tagliaferri E. (2002). Defibrotide as a promising treatment for thrombotic thrombocytopenic purpura in patients undergoing bone marrow transplantation. Bone Marrow Trans..

[133] Uderzo C., Fumagalli M., De Lorenzo P., Busca A., Vassallo E., Bonanomi S. (2000). Impact of thrombotic thrombocytopenic purpura on leukemic children undergoing bone marrow transplantation. Bone Marrow Trans..

[134] Schroder H. (1995). Defibrotide protects endothelial cells, but not L929 tumour cells, from tumour necrosis factor-alpha-mediated cytotoxicity. J. Pharm. Pharmacol..

[135] Corbacioglu S., Cesaro S., Faraci M., Valteau-Couanet D., Gruhn B., Rovelli A. (2012). Defibrotide for prophylaxis of hepatic veno-occlusive disease in paediatric haemopoietic stem-cell transplantation: an open-label, phase 3, randomised controlled trial. Lancet.

[136] Seaby E.G., Gilbert R.D. (2018). Thrombotic microangiopathy following haematopoietic stem cell transplant. Pediatr. Nephrol..

[137] Ostronoff F., Calixto R., Florencio R., Florencio M., Domingues M.C., Souto Maior A.P. (2007). Life-threatening hemolytic-uremic syndrome treated with rituximab in an allogeneic bone marrow transplant recipient. Bone Marrow Trans..

[138] Glezerman I.G., Jhaveri K.D., Watson T.H., Edwards A.M., Papadopoulos E.B., Young J.W. (2010). Chronic kidney disease, thrombotic microangiopathy, and hypertension following T Cell-depleted hematopoietic stem cell transplantation. Biol. Blood Marrow Trans..

[139] Bayer G., von Tokarski F., Thoreau B., Bauvois A., Barbet C., Cloarec S. (2019). Etiology and outcomes of thrombotic microangiopathies. Clin. J. Am. Soc. Nephrol..

[140] Lin Y.C., Chang H.K., Sun C.F., Shih L.Y. (1995). Microangiopathic hemolytic anemia as an initial presentation of metastatic cancer of unknown primary origin. South Med. J..

[141] Lechner K., Obermeier H.L. (2012). Cancer-related microangiopathic hemolytic anemia: clinical and laboratory features in 168 reported cases. Medicine (Baltimore).

[142] Rytting M., Worth L.f., N. (1996). Hemolytic disorders associated with cancer. Hematol Oncol Clin North Am..

[143] Oberic L., Buffet M., Schwarzinger M., Veyradier A., Clabault K., Malot S. (2009). Cancer awareness in atypical thrombotic microangiopathies. Oncologist.

[144] Francis K.K., Kalyanam N., Terrell D.R., Vesely S.K., George J.N. (2007). Disseminated malignancy misdiagnosed as thrombotic thrombocytopenic purpura: a report of 10 patients and a systematic review of published cases. Oncologist.

[145] Levi M., Ten Cate H. (1999). Disseminated intravascular coagulation. N. Engl. J. Med..

[146] Wada H., Matsumoto T., Suzuki K., Imai H., Katayama N., Iba T. (2018). Differences and similarities between disseminated intravascular coagulation and thrombotic microangiopathy. Thromb J..

[147] Ravindran A., Go R.S., Fervenza F.C., Sethi S. (2017). Thrombotic microangiopathy associated with monoclonal gammopathy. Kidney Int..

[148] Sethi S., Fervenza F.C., Rajkumar S.V. (2016). Spectrum of manifestations of monoclonal gammopathy-associated renal lesions. Curr. Opin. Nephrol. Hypertens.

[149] Yui J.C., Garceau D., Jhaveri K.D., Wanchoo R., Bijol V., Glezerman I. (2019). Monoclonal gammopathy-associated thrombotic microangiopathy. Am. J. Hematol..

